# Association between trace metals exposure and hearing loss

**DOI:** 10.3389/fpubh.2022.973832

**Published:** 2022-08-17

**Authors:** Peixi Zou, Menghuan Li, Wei Chen, Junfeng Ji, Fei Xue, Zhiyi Wang, Li Xu, You Cheng

**Affiliations:** ^1^Department of Otolaryngology-Head and Neck Surgery, Jinling Hospital, Medical School of Nanjing University, Nanjing, China; ^2^The First Clinical Medical College, Nanjing Medical University, Nanjing, China

**Keywords:** trace metals, exposure, health, hearing loss, association

## Abstract

**Background:**

Trace metals have side-effect on human health. The association between trace metals exposure and hearing loss remains unclear.

**Methods:**

A total of 8,128 participants were exacted for analysis of association between trace metals and hearing loss from the database of the National Health and Nutrition Examination Survey (NHANES) (2013–2018). Multivariable logistic regression and restricted cubic spline models were used to examine the association between trace metals and hearing loss.

**Results:**

Participants with hearing loss had a higher level of lead, cadmium, molybdenum, tin, thallium, and tungsten (all *p* < 0.05). After adjusting for confounders, compared with the reference of the lowest quartile, the ORs with 95%CIs for hearing loss across quartiles were 1.14 (0.86, 1.51), 1.49 (1.12, 1.98), 1.32 (0.97, 1.80) for cobalt, and 1.35 (0.98, 1.87), 1.58 (1.15, 2.16), 1.75 (1.28, 2.40) for tin. Individuals with the level of cobalt at third quartile had 49% higher risks of hearing loss than those at lowest quartile. And participants with highest quartile of tin had 1.75-folds risks of hearing loss than those with lowest quartile of tin. There were increasing trends in risks of hearing loss with a raised level of thallium (*p* for trend <0.05). Restricted cubic spline regression analysis indicated that there was a nonlinear association between hearing loss and the levels of tin (*p* for nonlinearity = 0.021). Subgroup analysis showed that individuals of female, without hypertension and diabetes, and with a higher level of low-density lipoprotein cholesterol had modified effects on the associations between hearing loss and exposure to tin.

**Conclusions:**

Our study indicated that exposure to cobalt and tin were significantly associated with hearing loss.

## Introduction

It is widely known that metals perform a critical function in the biochemical process. Essential trace elements (e.g., cobalt, iron, zinc, or copper) play a crucial role in enzyme synthesis, which regulate cellular homeostasis and energy production (1). Other elements such as lead, cadmium, tin, or mercury have an unknown impact on human body and are even toxic at certain levels (1, 2). Exposure to trace metals mainly occurred from our daily diet, inhalation, and dermal contact (1, 3). Source of lead can be released from batteries, pipes, pottery, and roofing materials, and cadmium can be touched by burning fossil fuels, cigarette smoke, and contaminated foods (4). Chronic or acute exposure to trace metals, even though the accumulation of essential elements at a high level, has a serious adverse effect on health (2, 5, 6). Excessive concentration of cobalt may lead to peripheral neuropathy, vision loss, sensorineural hearing loss, and cognitive decline (7). It was reported that early childhood exposure to lead may be a vital risk factor for hearing loss (8). Moreover, an epidemiologic study revealed that low-level exposure to cadmium was associated with hearing loss (9). Due to regeneration disabilities of the sensory receptor cells in the inner ear, the hearing loss induced by such toxicants is permanent (4).

Hearing loss is the fourth leading contributor to years lived with a disability worldwide (10). Various factors may contribute to hearing loss, including congenital, infectious, noise exposure, and aging. In clinical practice, hearing loss is defined as a pure-tone average exceeding 25dB (11). Several studies revealed that exposure to lead and cadmium could cause auditory dysfunction using 25dB as the threshold to identify hearing loss (9, 12, 13). But the grade of hearing conditions varied broadly, mild hearing loss may be reversible after correcting incentives or self-healing. It might be inappropriate to recklessly include all grades of hearing status to determine the association between hearing loss and heavy metals. Therefore, we extracted the data of hearing conditions with serious hearing difficulty from the National Health and Nutrition Examination Survey (NHANES), aiming to explore the relationship between severe hearing loss and urine trace metals.

## Methods

### Study population

The NHANES survey is a cross-sectional program recruiting a representative sample of participants from the United States population based on a complex, multistage probability design. The program collected data from demographic, clinical, and laboratory tests for surveyed individuals. The NHANES database can be acquired publicly from the Centers for Disease Control and Prevention National Center for Health Statistics (https://www.cdc.gov/nchs/nhanes/index.htm). Potential participants were exacted from the dataset of 2013–2018 (*N* = 29,400). Excluding those with unclear hearing conditions (*n* = 1,159), and missing data on urine trace metals (*n* = 20,113). A total of 8,128 participants were enrolled for further analysis. The survey protocol was approved by the NCHS research ethics review board, and all participants provided written informed consent. This study complied with the Declaration of Helsinki.

### Assessment of trace metals

Urine samples were measured for trace level element analysis by inductively coupled plasma mass spectrometry (ICP-MS) coupled with dynamic reaction cell technology (DRC). Liquid samples were introduced into the ICP through a nebulizer and spray chamber carried by a flowing argon stream, which ionizes the atoms. The ions are finally counted in rapid sequence at the detector allowing individual isotopes of an element to be determined. More detailed descriptions can be available at https://wwwn.cdc.gov/nchs/data/nhanes/2015-2016/labmethods/UTAS_UTASS_UM_UMS_I_MET.PDF. The current study included 10 trace metals elements (lead, barium, cadmium, cobalt, cesium, molybdenum, antimony, tin, thallium, and tungsten).

### Definition of hearing loss

Hearing loss in this study was defined as severe difficulty hearing. A household questionnaire on disability was provided to the surveyed participants by trained interviewers. Hearing loss was recorded if the person answered “yes” to the following question: are you deaf or do you have serious difficulty hearing.

### Covariates

Demographic data including age, gender, and race/ethnicity (Mexican American, other Hispanic, non-Hispanic white, non-Hispanic black, and other race) were collected from self-reports. As atherogenic risk factors are involved in the pathogenesis of hearing loss, covariates related to atherosclerosis were selected and controlled. Self-reported diabetes history, hypertension history, or hyperlipidemia history (yes/no), smoker (smoked at least 100 cigarettes in life), and alcohol user (at least 12 alcoholic drinks per year or not) were documented from questionnaire data. Noise exposure was defined as “ever exposed to very loud noise at work.” The level of triglyceride, total cholesterol, low-density lipoprotein cholesterol, and urine trace metals were obtained from laboratory data.

### Statistical analysis

Data distribution was determined by the Kolmogorov-Smirnov test. Continuous data with normal distribution were presented as mean ± deviation and were analyzed with independent sample student's *t*-test. Nonnormal distributed continuous data were reported as median with interquartile range (IQR) and compared by nonparametric Mann–Whitney *U*-test. Categorical variables were calculated with counts and percentages and were assessed by the Chi-square test. Log2-transform of trace metals was performed to facilitate the interpretation due to its skewed distribution.

Multivariate logistic regression analysis was used to assess the association of trace metals quartiles with hearing loss. Model 1 was adjusted for age and gender. Model 2 was adjusted for age, gender, race, hypertension, diabetes, hyperlipidemia, and noise exposure. And model 3 was adjusted for age, gender, race, hypertension, diabetes, hyperlipidemia, noise exposure, triglyceride, total cholesterol, and low-density lipoprotein cholesterol. In order to explore the effect of multiple-metal on hearing loss, we conducted a collinearity diagnostic to determine whether there are collinearities among these metals. Variance inflation factors (VIF) and tolerance are used to identify a collinearity (Tolerance<0.1 or VIF>10 is considered to be collinear). We include ten trace metals into covariates when they are non-collinear, as well as the covariates in the model 3 to build a multiple-metal based model. The odds ratio (OR) with 95% confidence intervals (CI) was calculated.

A restricted cubic spline regression model was applied to explore any nonlinear relationship between trace metals and hearing loss [with 3 knots located at 10th, 50th, and 90th percentiles confirmed according to the Akaike information criterion (14)]. Subgroup analyses stratified by age, gender, hypertension, diabetes, and level of low-density lipoprotein cholesterol were conducted to determine the associations between trace metals and hearing loss. All statistical analyses were performed by R software (version 4.0.5) and SPSS software (version 25). Two-sided *p* < 0.05 was considered statistically significant.

## Results

### Baseline characteristics

A total of 8,128 participants were included in this study, with a prevalence of hearing loss of approximately 5.5%. There were significant differences between the hearing group and non-hearing group in age, gender, race/ethnicity, hypertension, diabetes, hyperlipidemia, noise exposure, levels of triglyceride, total cholesterol, and low-density lipoprotein cholesterol (All *p* < 0.05). As for the concentration of urine trace metals, the hearing loss group had a higher level of lead, cadmium, molybdenum, tin, thallium, and tungsten (All *p* < 0.05). The general presentation of baseline characteristics was shown in Table 1.

**Table 1:** Baseline characteristics of the study population.

**Variables**	**Overall (*N* = 8,128)**	**Hearing loss (*N1* = 449)**	**Non-hearing loss (*N2* = 7,679)**	***P* value**
Age, years	34 [14–57]	67 [55–79]	32 [14–55]	<0.001
Male, %	4,035 (49.6)	279 (62.1)	3,756 (48.9)	<0.001
Race/ethnicity, %				<0.001
Mexican American	1,420 (17.5)	75 (16.7)	1,345 (17.5)	
Other Hispanic	853 (10.5)	48 (10.7)	805 (10.5)	
Non-Hispanic white	2,687 (33.1)	227 (50.6)	2,460 (32.0)	
Non-Hispanic Black	1,806 (22.2)	55 (12.2)	1,751 (22.8)	
Other race	1,362 (16.8)	44 (9.8)	1,318 (17.2)	
Hypertension, %	2,035 (25.0)	240 (53.5)	1,795 (23.4)	<0.001
Diabetes, %	781 (9.6)	114 (25.4)	667 (8.7)	<0.001
Hyperlipidemia, %	1,978 (24.3)	224 (49.9)	1,754 (22.8)	<0.001
Smoker, %	1,606 (19.8)	97 (21.6)	1,509 (19.7)	0.313
Alcohol user, %	6,086 (74.9)	352 (78.4)	5,734 (74.7)	0.077
Triglyceride, mmol/L	1.21 ± 1.13	1.32 ± 1.08	1.20 ± 1.13	0.033
LDL-C, mmol/L	2.72 ± 0.89	2.83 ± 0.92	2.71 ± 0.88	0.008
Total Cholesterol, mmol/L	4.63 ± 1.04	4.79 ± 1.07	4.61 ± 1.04	0.001
Noise exposure	4,827 (59.4)	320 (71.3)	4,507 (58.7)	<0.001
Trace metals				
Lead, ug/L	0.3 [0.16–0.53]	0.42 [0.23–0.76]	0.29 [0.16–0.52]	<0.001
Barium, ug/L	1.05 [0.51–2.09]	0.96 [0.48–1.94]	1.06 [0.51–2.11]	0.102
Cadmium, ug/L	0.12 [0.05–0.29]	0.28 [0.13–0.52]	0.11 [0.05–0.27]	<0.001
Cobalt, ug/L	0.44 [0.25–0.71]	0.43 [0.26–0.65]	0.45 [0.25–0.72]	0.340
Cesium, ug/L	4.40 [2.60–6.60]	4.29 [2.79–6.29]	4.41 [2.58–6.62]	0.680
Molybdenum, ug/L	42.34 [21.4–74.93]	38.00 [20.84–64.95]	42.68 [21.51–75.70]	0.008
Antimony, ug/L	0.05 [0.03–0.09]	0.48 [0.03–0.08]	0.05 [0.03–0.09]	0.437
Tin, ug/L	0.52 [0.23–1.24]	0.70 [0.35–1.48]	0.50 [0.22–1.22]	<0.001
Thallium, ug/L	0.17 [0.10–0.26]	0.14 [0.08–0.21]	0.17 [0.10–0.26]	<0.001
Tungsten, ug/L	0.07 [0.03–0.15]	0.05 [0.03–0.10]	0.08 [0.03–0.16]	<0.001

*LDL-C, low-density lipoprotein cholesterol*.

### Association between trace metals and hearing loss

**Figure 1:**
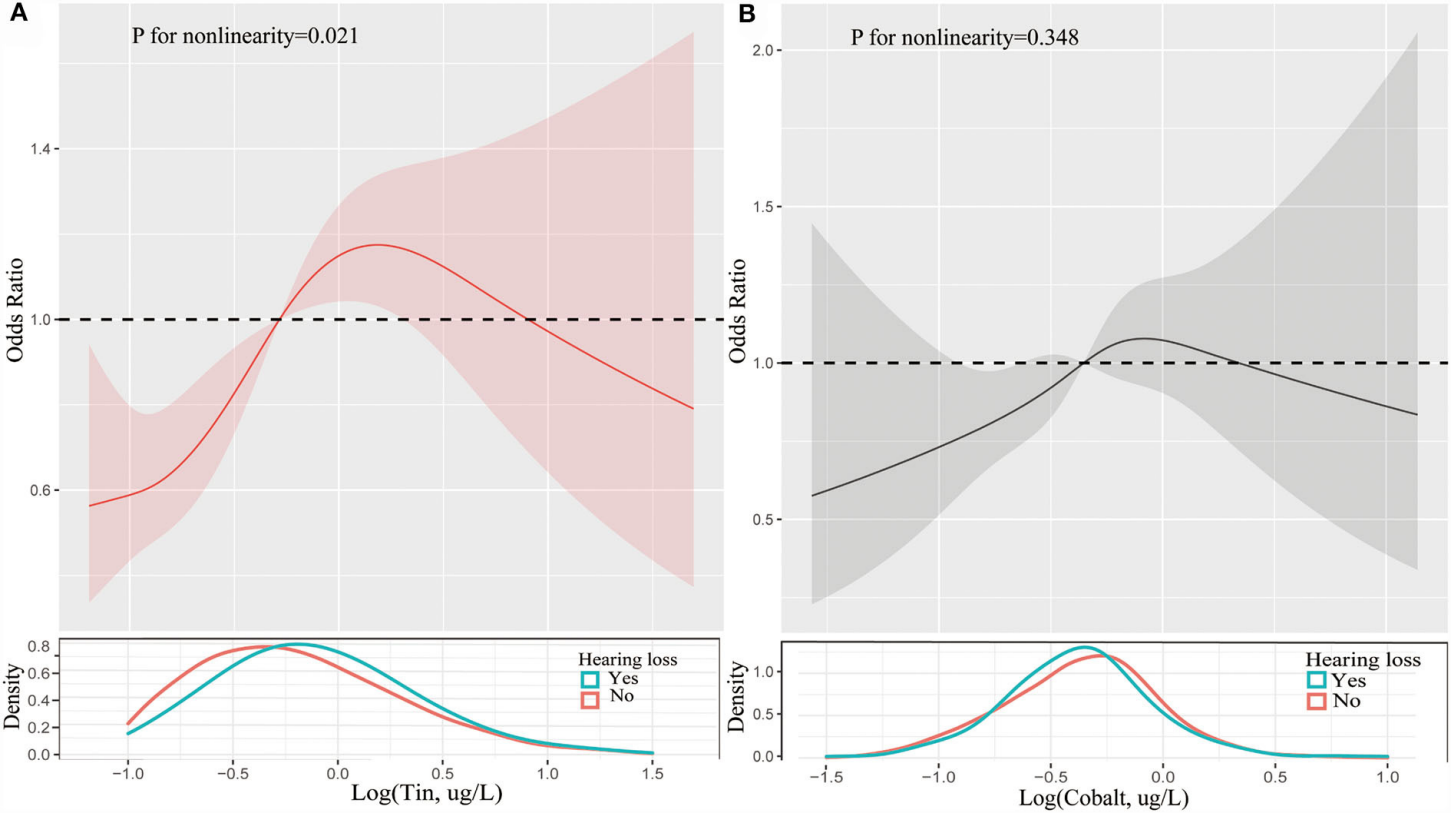
Restricted cubic spline of association between the levels of tin **(A)** and cobalt **(B)** and the risks of hearing loss. Analyses were adjusted for age, gender, race, hypertension, diabetes, hyperlipidemia, noise exposure, triglyceride, total cholesterol and low-density lipoprotein cholesterol. The solid line and dashed line represent the log-transformed odds ratios and corresponding 95% confidence intervals.

Table 2 presented the results of logistic regression analyses for the association between trace metals and hearing loss. When fully adjusted for potential confounders, compared with the reference of lowest quartile, the ORs with 95%CIs for hearing loss across highest quartile was 1.10 (0.81, 1.49) for lead, 1.22 (0.91, 1.64) for barium, 1.04 (0.68, 1.58) for cadmium, 1.32 (0.97, 1.80) for cobalt, 0.90 (0.67, 1.23) for cesium, 0.95 (0.70, 1.30) for molybdenum, 1.17 (0.87, 1.58) for antimony, 1.75 (1.28, 2.40) for tin, 0.83 (0.61, 1.14) for thallium, and 0.95 (0.69, 1.30) for tungsten, respectively. Cobalt, and tin were significantly associated with hearing loss (*p* < 0.05). Individuals with the level of cobalt at third quartile had 49% higher risks of hearing loss than those at lowest quartile. And participants with highest quartile of tin had 1.75-folds risks of hearing loss than those with lowest quartile of tin. Restricted cubic spline indicated that there was a nonlinear association between hearing loss and the levels of tin (*p* for nonlinearity = 0.021, Figure 1A). However, the association between the risk of hearing loss and levels of cobalt was not nonlinear (*p* for nonlinearity > 0.05, Figure 1B). We also conducted multiple-metal based Cox-regression analyses including 10 trace metals as covariates (Table S1). It showed that higher level of lead, cadmium, tin, thallium, and tungsten were statistically associated with hearing loss in an unadjusted model. After adjusting for all confounders, higher tin concentration retained higher risk of hearing loss. The highest quartile of tin had 2.09-folds risks for hearing loss compared with the lowest quartile. And cobalt was also closely related to the occurrence of hearing loss in the full adjusted model.

**Table 2:** Odds ratio for the association between urine trace metals and hearing loss.

**Metals**	**Model 1**		**Model 2**		**Model 3**	
	**OR (95% CI)**	**P-t**	**OR (95% CI)**	**P-t**	**OR (95% CI)**	**P-t**
Lead		0.613		0.579		0.609
Q1	Ref.		Ref.		Ref.	
Q2	0.93 (0.67, 1.29)		0.94 (0.67, 1.30)		0.93 (0.66, 1.29)	
Q3	0.95 (0.69, 1.31)		0.98 (0.71, 1.35)		0.96 (0.70, 1.33)	
Q4	1.10 (0.81, 1.48)		1.12 (0.83, 1.52)		1.10 (0.81, 1.49)	
Barium		0.644		0.431		0.445
Q1	Ref.		Ref.		Ref.	
Q2	1.06 (0.80, 1.39)		1.10 (0.83, 1.45)		1.10 (0.83, 1.45)	
Q3	1.18 (0.89, 1.56)		1.23 (0.93, 1.64)		1.23 (0.92, 1.63)	
Q4	1.15 (0.86, 1.54)		1.22 (0.91, 1.64)		1.22 (0.91, 1.64)	
Cadmium		0.172		0.078		0.094
Q1	Ref.		Ref.		Ref.	
Q2	0.91 (0.59, 1.42)		0.91 (0.58, 1.42)		0.91 (0.58, 1.42)	
Q3	0.77 (0.50, 1.19)		0.77 (0.50, 1.19)		0.76 (0.50, 1.17)	
Q4	1.01 (0.67, 1.53)		1.06 (0.70, 1.62)		1.04 (0.68, 1.58)	
Cobalt		0.015		0.028		0.037
Q1	Ref.		Ref.		Ref.	
Q2	1.19 (0.90, 1.57)		1.16 (0.88, 1.54)		1.14 (0.86, 1.51)	
Q3	1.56 (1.17, 2.06) ^**^		1.51 (1.14, 2.01) ^**^		1.49 (1.12, 1.98) ^**^	
Q4	1.38 (1.02, 1.88) ^*^		1.35 (0.99, 1.83)		1.32 (0.97, 1.80)	
Cesium		0.334		0.356		0.306
Q1	Ref.		Ref.		Ref.	
Q2	1.18 (0.89, 1.56)		1.20 (0.90, 1.59)		1.19 (0.89, 1.57)	
Q3	1.06 (0.79, 1.41)		1.05 (0.79, 1.41)		1.03 (0.77, 1.38)	
Q4	0.91 (0.67, 1.23)		0.94 (0.69, 1.27)		0.90 (0.67, 1.23)	
Molybdenum		0.772		0.866		0.870
Q1	Ref.		Ref.		Ref.	
Q2	1.14 (0.87, 1.49)		1.01 (0.84, 1.44)		1.08 (0.82, 1.41)	
Q3	1.10 (0.83, 1.45)		1.05 (0.80, 1.40)		1.03 (0.78, 1.36)	
Q4	1.03 (0.76, 1.39)		0.99 (0.73, 1.34)		0.95 (0.70, 1.30)	
Antimony		0.290		0.495		0.592
Q1	Ref.		Ref.		Ref.	
Q2	1.03 (0.78, 1.36)		1.01 (0.76, 1.34)		1.00 (0.75, 1.32)	
Q3	1.22 (0.92, 1.61)		1.17 (0.88, 1.55)		1.14 (0.86, 1.51)	
Q4	1.26 (0.94, 1.69)		1.19 (0.89, 1.61)		1.17 (0.87, 1.58)	
Tin		0.001		0.002		0.004
Q1	Ref.		Ref.		Ref.	
Q2	1.42 (1.03, 1.96) ^*^		1.37 (0.99, 1.89)		1.35 (0.98, 1.87)	
Q3	1.69 (1.23, 2.30) ^**^		1.59 (1.17, 2.18) ^**^		1.58 (1.15, 2.16) ^**^	
Q4	1.88 (1.38, 2.57) ^***^		1.79 (1.31, 2.45) ^***^		1.75 (1.28, 2.40) ^***^	
Thallium		0.006		0.011		0.009
Q1	Ref.		Ref.		Ref.	
Q2	1.20 (0.93, 1.55)		1.23 (0.95, 1.59)		1.22 (0.94, 1.57)	
Q3	0.77 (0.58, 1.03)		0.79 (0.59, 1.05)		0.77 (0.58, 1.03)	
Q4	0.79 (0.58, 1.07)		0.86 (0.63, 1.17)		0.83 (0.61, 1.14)	
Tungsten		0.806		0.771		0.706
Q1	Ref.		Ref.		Ref.	
Q2	1.10 (0.85, 1.42)		1.05 (0.81, 1.36)		1.04 (0.80, 1.35)	
Q3	0.96 (0.73, 1.26)		0.91 (0.69, 1.19)		0.89 (0.67, 1.17)	
Q4	1.00 (0.73, 1.37)		0.97 (0.70, 1.33)		0.95 (0.69, 1.30)	

*Adjusted covariates in model 1 were age and gender; Adjusted covariates in model 2 were age, gender, race, hypertension, diabetes, hyperlipidemia, and noise exposure; Adjusted covariates in model 3 were age, gender, race, hypertension, diabetes, hyperlipidemia, noise exposure, triglyceride, total cholesterol, and low-density lipoprotein cholesterol. P-t, p for trend*.

* *P < 0.05*,

** *P < 0.01*,

*** *P < 0.001; OR, odds ratio; CI, confident interval*.

### Subgroup analysis

When stratified by age, gender, hypertension, diabetes, and the level of low-density lipoprotein cholesterol, we determined the effect size of these factors on hearing loss. As shown in Table 3, individuals of female, without hypertension and diabetes, and with a higher level of LDL-C had modified effects on the association between hearing loss and exposure to tin. Similarly, compared with reference of lowest quartile, participants of female still had higher risks of hearing loss when exposed to higher level of cobalt (OR 2.27, 95% CI 1.44–3.58). Interestingly, participants with a lower level of LDL-C were more likely to have hearing loss in the third quartile of cobalt (OR 1.88, 95%CI 1.21–2.92).

**Table 3:** Subgroup analysis of the association between urine trace metals and hearing loss.

**Subgroup**	**Q1**	**Q2**	**Q3**	**Q4**	**P-t**	**P-int**
		**OR (95%CI)**	**OR (95%CI)**	**OR (95%CI)**		
Tin						
Age						0.270
<65 years	Ref.	1.55 (1.02, 2.37) ^*^	1.66 (1.09, 2.53) ^*^	1.36 (0.87, 2.13)	0.097	
≥65 years	Ref.	1.16 (0.70, 1.91)	1.35 (0.85, 2.16)	1.81 (1.14, 2.87) ^*^	0.034	
Gender						0.407
Male	Ref.	1.14 (0.76, 1.72)	1.32 (0.89, 1.96)	1.37 (0.91, 2.06)	0.403	
Female	Ref.	1.74 (1.01, 2.99) ^*^	2.04 (1.21, 3.45) ^**^	2.44 (1.46, 4.09) ^***^	0.007	
Hypertension						0.700
Yes	Ref.	1.44 (0.89, 2.35)	1.47 (0.91, 2.35)	1.56 (0.97, 2.51)	0.318	
No	Ref.	1.20 (0.77, 1.86)	1.76 (1.09, 2.54) ^*^	1.89 (1.24, 2.90) ^**^	0.008	
Diabetes						0.220
Yes	Ref.	1.49 (0.74, 3.03)	1.37 (0.69, 2.70)	1.10 (0.56, 2.18)	0.594	
No	Ref.	1.31 (0.91, 1.89)	1.61 (1.13, 2.30) ^**^	2.01 (1.41, 2.86) ^***^	0.001	
LDL-C						0.724
Below median (<2.64 mmol/L)	Ref.	1.68 (1.00, 2.82)	1.81 (1.10, 2.98) ^*^	1.88 (1.14, 3.10) ^*^	0.079	
Above median (≥2.64 mmol/L)	Ref.	1.17 (0.77, 1.78)	1.44 (0.96, 2.16)	1.74 (1.16, 2.62) ^**^	0.036	
Cobalt						
Age						0.097
<65 years	Ref.	1.14 (0.77, 1.70)	1.21 (0.82, 1.79)	0.84 (0.54, 1.32)	0.374	
≥65 years	Ref.	1.13 (0.76, 1.67)	1.55 (1.03, 2.32) ^*^	1.52 (0.99, 2.34)	0.086	
Gender						0.117
Male	Ref.	1.00 (0.70, 1.44)	1.14 (0.79, 1.64)	1.14 (0.76, 1.70)	0.822	
Female	Ref.	1.37 (0.87, 2.17)	2.27 (1.44, 3.58) ^***^	1.62 (0.99, 2.66)	0.003	
Hypertension						0.104
Yes	Ref.	1.07 (0.73, 1.58)	1.39 (0.92, 2.08)	1.68 (1.11, 2.56) ^*^	0.052	
No	Ref.	1.23 (0.81, 1.84)	1.59 (1.06, 2.36) ^*^	0.98 (0.61, 1.57)	0.069	
Diabetes						0.818
Yes	Ref.	0.99 (0.56, 1.77)	1.71 (0.94, 3.13)	1.40 (0.73, 2.70)	0.196	
No	Ref.	1.18 (0.85, 1.63)	1.43 (1.04, 1.98) ^*^	1.29 (0.91, 1.84)	0.170	
LDL-C						0.371
Below median (<2.64 mmol/L)	Ref.	1.32 (0.85, 2.03)	1.88 (1.21, 2.92) ^**^	1.25 (0.77, 2.03)	0.039	
Above median (≥2.64 mmol/L)	Ref.	1.06 (0.73, 1.53)	1.28 (0.88, 1.86)	1.40 (0.94, 2.09)	0.293	

*The analysis was adjusted for age, gender, race, hypertension, diabetes, hyperlipidemia, noise exposure, triglyceride, total cholesterol, and low-density lipoprotein cholesterol. LDL-C, low-density lipoprotein cholesterol. OR, odds ratio; CI, confident interval. P-t, p for trend*.

* *P < 0.05*,

** *P < 0.01*,

*** *P < 0.001. P-int, p for interaction*.

## Discussion

This is the first study that implicated the association between trace metals and severe hearing loss. There are three main findings of the current study. Firstly, we highlighted the role of atherogenic risk factors (aging, hypertension, diabetes, hyperlipidemia, et. al) in the prevalence of hearing loss. Secondly, tin was firstly found to be significantly associated with hearing loss in a large sample cross-sectional study. Lastly, the trends of association between hearing loss and the level of tin were nonlinear.

Hearing loss affects individuals worldwide with nearly 6%-8% of the world population (10). In our study, we reported the prevalence of hearing loss of 5.5% based on a representative sample, which was closed to the above literature. Traditional risk factors for hearing loss were infectious, noise exposure, and aging (10). It was demonstrated that atherosclerosis may influence hearing loss (15), so we compared the difference in atherogenic risk factors between the population with hearing loss and without hearing loss. Consistently, the proportions of hypertension, diabetes, hyperlipidemia, and the level of blood lipid profile were significantly higher in the population with hearing loss. The above comorbidities were well confirmed in the pathophysiological process of atherosclerosis and were independent risk factors for coronary artery disease and stroke. These comorbidities might contribute to sclerosis of the blood vessels that supply hearing organs, leading to limited blood flow and declining hearing function.

A series of case reports showed that systemic cobalt toxicity was closed related to peripheral neuropathy, vision loss, sensorineural hearing loss, and cognitive decline (6, 7, 12). Rizzetti et al. reported a case of hearing and vision loss after hip-arthroplasty, which contained cobalt, with increased concentration of cobalt in urine and blood samples (7). Cobalt can produce various toxicological effects including local respiratory toxicity, neurological toxicity, and immunological toxicity (7). Cobalt can induce a hypoxia-like effect, possibly targeting mitochondria (7) and impairing nerve function, with patients presenting hearing loss. An experimental study using rabbits to explore its pathological alterations after repeated intravenous injection of cobalt indicated clinical signs indicative of auditory and optic system toxicity (16). In addition, histopathological examination showed severe retinal and cochlear ganglion cell depletion along with optic nerve damage and loss of sensory cochlear hair cells in these experimental rabbits (16). This evidence could further confirm the neurological toxic effect of cobalt. It was reported that higher level of tin in individuals with hearing frustration (17). And we also determined that cobalt is closely associated with hearing loss from the perspective of epidemiological study. Continuous cobalt exposure mainly concerning workers exposed in occupational setting is deemed to be detrimental to health.

Tin is a naturally existing element, which is found in inorganic and organic forms in the environment. Exposure to tin and tin compounds in the U.S. population is almost ubiquitous (9). Tin levels in the body increase with age in adult (9). World Health Organization classified the tin as potentially toxic (18). Studies investigated that indium-tin-oxide could cause pulmonary and systemic toxicity in rat models (19). It also demonstrated that elevated tin level was associated with increased fasting glucose level in coke oven workers in China (20). Shiue et al. reported that the level of tin was higher in hearing frustration population (17), which was consistent with our results. However, experimental investigations for the links of tin to auditory dysfunction are less conducted, mechanism of tin-induced hearing loss remained unclear.

Heavy metals are particular chemical class of elements and present in the environment. Most of them are harmful even at low concentrations. Several potential mechanisms are implicated in metals toxicity including production of oxygen reactive species (ROS), interaction with thiol groups of proteins, incorrect protein folding and mimicry of the essential elements for intracellular transport, and depletion of antioxidants enzymes (1). Cadmium is capable to bypass the blood-brain barrier (21). On the other hand, in human autopsies of individuals with diverse neurodegenerative diseases, significantly higher level of accumulation of Cd in locus ceruleus was observed (22). Similarly, tin as a heavy metal element, plays a critical role in neurological disease and autoimmune disease (23, 24). Researchers and in our study, tin was dramatically associated with hearing loss. We assumed that it impaired hearing through the destruction of neurons. Further cellular and animal research is prompted for verification.

There are several limitations of our study. Firstly, a cross-sectional study design can only infer the correlation, but causality. Secondly, despite the attempts to adjust hearing-related confounders, unmeasured or unknown covariates may influence the effectiveness of the conclusion. Additionally, the mechanism of the association between hearing loss and trace metals has not been fully addressed. Finally, the data of this study was derived from the United state, the generalizability of the conclusion to other regions or populations should be very cautious.

## Conclusions

Our study indicated that exposure to cobalt and tin were significantly associated with hearing loss.

## Data availability statement

The datasets presented in this study can be found in online repositories. The names of the repository/repositories and accession number(s) can be found below: https://www.cdc.gov/nchs/nhanes/index.htm.

## Ethics statement

The studies involving human participants were reviewed and the survey protocol was approved by the NCHS research ethics review board. Written informed consent to participate in this study was provided by the participants' legal guardian/next of kin.

## Author contributions

PZ: conceptualization, methodology, and writing-original draft. ML: data curation, software, formal analysis, and writing-review and editing. WC: writing-original draft. JJ, FX, and LX: investigation. YC: validation. All authors contributed to the article and approved the submitted version.

## Funding

This work was supported by the Strengthening Health through Science and Education Program of Nanjing Commission of Health (SZDZK202002). The funding body played no role in the design of the study and collection, analysis, and interpretation of data, and in writing the manuscript.

## Conflict of interest

The authors declare that the research was conducted in the absence of any commercial or financial relationships that could be construed as a potential conflict of interest.

## Publisher's note

All claims expressed in this article are solely those of the authors and do not necessarily represent those of their affiliated organizations, or those of the publisher, the editors and the reviewers. Any product that may be evaluated in this article, or claim that may be made by its manufacturer, is not guaranteed or endorsed by the publisher.
