# Supplementary material for: Association between trace metals exposure and hearing loss
Source: Front Public Health. 2022 Aug 17;10:973832. doi: 10.3389/fpubh.2022.973832 (PMC9428401; doi:10.3389/fpubh.2022.973832)
Supplement: Supplementary file 1 [file Table_1.DOCX]

Table S1. Multiple-elements based logistic regression model for assessing the association between trace metals and hearing loss.

| Variables | Unadjusted model | | Adjusted model | |
| --- | --- | --- | --- | --- |
|  | OR (95% CI) | P-t | OR (95% CI) | P-t |
| Lead |  | <0.001 |  | 0.779 |
| Q1 | Ref. |  | Ref. |  |
| Q2 | 1.26 (0.92-1.72) |  | 0.86 (0.60-1.25) |  |
| Q3 | 1.66 (1.22-2.26)** |  | 0.86 (0.58-1.27) |  |
| Q4 | 2.64 (2.00-3.50)*** |  | 0.94 (0.62-1.42) |  |
| Barium |  | 0.450 |  | 0.978 |
| Q1 | Ref. |  | Ref. |  |
| Q2 | 0.95 (0.73-1.24) |  | 0.98 (0.73-1.31) |  |
| Q3 | 0.91 (0.70-1.19) |  | 1.02 (0.75-1.41) |  |
| Q4 | 0.80 (0.61-1.06) |  | 0.96 (0.68-1.36) |  |
| Cadmium |  | <0.001 |  | 0.146 |
| Q1 | Ref. |  | Ref. |  |
| Q2 | 2.35 (1.55-3.56)*** |  | 0.98 (0.62-1.56) |  |
| Q3 | 3.95 (2.68-5.84)*** |  | 0.88 (0.54-1.37) |  |
| Q4 | 7.44 (5.13-10.79)*** |  | 1.17 (0.71-1.91) |  |
| Cobalt |  | 0.015 |  | 0.029 |
| Q1 | Ref. |  | Ref. |  |
| Q2 | 1.28 (0.98-1.68) |  | 1.27 (0.91-1.77) |  |
| Q3 | 1.27 (0.97-1.66) |  | 1.75 (1.20-2.56)** |  |
| Q4 | 0.88 (0.66-1.17) |  | 1.53 (1.01-2.33)* |  |
| Cesium |  | 0.010 |  | 0.283 |
| Q1 | Ref. |  | Ref. |  |
| Q2 | 1.45 (1.11-1.89)** |  | 0.99 (0.70-1.39) |  |
| Q3 | 1.25 (0.95-1.65) |  | 0.81 (0.53-1.22) |  |
| Q4 | 0.98 (0.73-1.31) |  | 0.69 (0.43-1.11) |  |
| Molybdenum |  | 0.005 |  | 0.959 |
| Q1 | Ref. |  | Ref. |  |
| Q2 | 1.13 (0.87-1.45) |  | 1.00 (0.74-1.36) |  |
| Q3 | 0.97 (0.74-1.26) |  | 1.00 (0.70-1.42) |  |
| Q4 | 0.68 (0.51-0.90)** |  | 0.92 (0.61-1.38) |  |
| Antimony |  | 0.269 |  | 0.700 |
| Q1 | Ref. |  | Ref. |  |
| Q2 | 1.08 (0.83-1.41) |  | 0.96 (0.70-1.31) |  |
| Q3 | 1.10 (0.84-1.43) |  | 1.10 (0.78-1.55) |  |
| Q4 | 0.85 (0.65-1.12) |  | 1.15 (0.79-1.68) |  |
| Tin |  | <0.001 |  | 0.001 |
| Q1 | Ref. |  | Ref. |  |
| Q2 | 1.69 (1.24-2.30)** |  | 1.42 (1.08-2.15)* |  |
| Q3 | 2.10 (1.56-2.83)*** |  | 1.88 (1.32-2.68)*** |  |
| Q4 | 2.17 (1.62-2.93)*** |  | 2.09 (1.45-3.02)*** |  |
| Thallium |  | <0.001 |  | 0.012 |
| Q1 | Ref. |  | Ref. |  |
| Q2 | 1.11 (0.88-1.42) |  | 1.03 (0.76-1.40) |  |
| Q3 | 0.65 (0.49-0.85)** |  | 0.64 (0.44-0.92)* |  |
| Q4 | 0.49 (0.37-0.66)*** |  | 0.69 (0.45-1.06) |  |
| Tungsten |  |  |  | 0.322 |
| Q1 | Ref. | <0.001 | Ref. |  |
| Q2 | 1.00 (0.79-1.28) |  | 0.93 (0.69-1.24) |  |
| Q3 | 0.71 (0.55-0.93)* |  | 0.75 (0.54-1.05) |  |
| Q4 | 0.47 (0.35-0.63)*** |  | 0.77 (0.52-1.14) |  |

The model was adjusted for age, gender, race, hypertension, diabetes, hyperlipidemia, noise exposure, triglyceride, total cholesterol, low-density lipoprotein cholesterol, and all trace metals. *P<0.05, **P<0.01, ***P<0.001; OR, odds ratio; CI, confident interval.
